# Supplementary material for: Implementation of the Community Assets Supporting Transitions (CAST) transitional care intervention for older adults with multimorbidity and depressive symptoms: A qualitative descriptive study
Source: PLoS One. 2022 Aug 5;17(8):e0271500. doi: 10.1371/journal.pone.0271500 (PMC9355229; doi:10.1371/journal.pone.0271500)
Supplement: S1 Appendix — (DOCX) [file pone.0271500.s001.docx]

**S1 Appendix. Meeting Minutes Reviewed for Document Analysis**

| **Project Meeting Minutes/Notes** | **Number** |
| --- | --- |
| Introductory and Strategy Meetings | 53 |
| Community Advisory Board Meetings | 13 |
| Care Transition Coordinator Meetings | 20 |
| Research Coordinator notes in relation to internal research team meetings and recruiter meetings (125)/ Investigator meetings (4) | 130 |
| Miscellaneous Meetings | 17 |
| **Total Reviewed** | 233 |
