# Supplementary material for: Implementation of the Community Assets Supporting Transitions (CAST) transitional care intervention for older adults with multimorbidity and depressive symptoms: A qualitative descriptive study
Source: PLoS One. 2022 Aug 5;17(8):e0271500. doi: 10.1371/journal.pone.0271500 (PMC9355229; doi:10.1371/journal.pone.0271500)
Supplement: S2 Appendix — (DOCX) [file pone.0271500.s002.docx]

**S2 Appendix. Interview Guides for Care Transition Coordinators**

**Time point 1:**

Thank you for taking the time to participate in our interview. I just want to start by giving you a bit of an introduction to what we will be talking about today. Today we’re going to be talking about the approaches used to plan and conduct the Community Assets Supporting Transitions (CAST) study. We are interested in how the program has been adapted to meet the needs of communities and what will need to happen to continue the program after the study is complete.

When we’re talking about conducting the research, we are referring to the many different stages of CAST’s work in which you may have participated or been engaged, from the shaping how the study should roll out, to recruitment strategies, and collecting data, and so on.

With these ideas in mind, we’d like to start with some questions about the CAST program.

1. What kind of training was provided for you?
   - Do you feel the training has prepared you to carry out the roles and responsibilities expected of you? Can you explain?
   - What were the positive aspects of the training?
   - What was missing or was not provided in sufficient detail? Is there anything we should clarify or include in training for others in the future?
   - What kind of continued training is planned? What additional training would be useful to support you in your role?
   - Who do you ask if you have questions about the intervention or its implementation? How available are these individuals?
2. What kinds of information and materials about the intervention were made available to you?
   - Copies of materials?
   - Personal contact?
   - Internal information sharing; e.g., staff meetings?
   - Were the information and materials timely? Relevant? Sufficient?
3. Can you describe what support you have had by the research team and the extent to which this support met your needs?
   - Can you describe a recent example?
4. How confident are you that you will be able to successfully implement the intervention?
   - What gives you that level of confidence (or lack of confidence)?
5. Has the intervention been implemented according to the implementation plan? Please tell me about both successes and challenges.
   - Based on your experiences to date: how well have you been able to ensure that your care is person- and family-centred?
   - Probes: e.g., goal-centred, looked at the whole person, tailoring, partnering
6. Based on your experiences thus far, what kinds of changes or alterations have you needed to make to the intervention to work effectively in your community?
7. What kinds of information and materials about the intervention are provided for individuals in your community (e.g., other service providers, participants)?
   - Copies of materials?
   - Personal contact?
   - Internal information sharing; e.g., study-related meetings?
   - Is the information relevant? Sufficient?
8. Based on your experiences implementing the CAST study to date, who are influential community members that have been important to get on board with the intervention? How have you gotten them on board?
9. Can you describe your working relationships with your community-based colleagues when implementing the intervention?
   - With colleagues in your organization?*(**only ask if RN-CTC is hired through an external agency/organization [i.e., via secondment])*
   - With colleagues in other organizations?
   - Can you tell me a story about a time you needed to work with others to solve a problem? Or to implement this intervention?
10. What steps have you taken to encourage other community providers to commit to engaging with you to support the intervention?
    - Which individuals have you targeted?
    - How have you approached them?
    - What information have you given them?
    - How frequently and how do you communicate with them?
11. How complicated is the CAST intervention?
    - Please consider the following aspects of the CAST intervention: duration, scope, intricacy, and number of steps involved and whether the intervention reflects a clear departure from previous practices.
12. What components of the CAST intervention have been implemented most successfully to date?
    - care coordination, system navigation and case management (direct or indirect)
    - clinical assessments and screening
    - health education and health literacy
    - behavioural change support (e.g., behavioural activation, problem-solving therapy, supporting behaviour change)
    - clinical care (e.g., management of chronic conditions and depressive symptoms, includes medication reconciliation, review and management)
    - psychosocial and practical support
    - building trusting patient-caregiver-provider relationships
    - health promotion and disease prevention
    - self-management support
    - caregiver health and support
13. What components of the CAST intervention have been most challenging to implement?
    - care coordination, system navigation and case management (direct or indirect)
    - clinical assessments and screening
    - health education and health literacy
    - behavioural change support (e.g., behavioural activation, problem-solving therapy, supporting behaviour change)
    - clinical care (e.g., management of chronic conditions and depressive symptoms, includes medication reconciliation, review and management)
    - psychosocial and practical support
    - building trusting patient-caregiver-provider relationships
    - health promotion and disease prevention
    - self-management support
    - caregiver health and support
14. What challenges have you faced connecting with or working with participants in implementing the intervention? How have you tried to address these?
15. Can you give an example of when the program made a difference to an older adult participant?
16. Can you give an example of when the program made a difference to a caregiver?
17. Do you have any additional comments or feedback on the implementation of the CAST study and your role to date that we have not yet discussed and you would like to add?

**Time point 2:**

Thank you for taking the time to participate in our interview. I just want to start by giving you a bit of an introduction to what we will be talking about today. Today we’re going to be talking about the approaches used to plan and conduct Community Assets Supporting Transitions (CAST) study. We are interested in how the program has been adapted to meet the needs of communities and what will need to happen to continue the program after the study is complete.

When we’re talking about conducting the research, we are referring to the many different stages of CASTs work in which you may have participated or been engaged, from the shaping how the study should roll out, to recruitment strategies, and collecting data, and so on.

Now that you’ve been implementing your role for some time, we’d like to start with revisiting some questions about the CAST program.

- - 1. Can you describe what support you have had by the research team and the extent to which this support met your needs?
  - Can you describe a recent example?

1. How confident are you that you have been able to successfully implement the intervention?
   - What gives you that level of confidence (or lack of confidence)?
2. Has the intervention been implemented according to the implementation plan? Please tell me about both successes and challenges.
   - Based on your experiences to date: how well have you been able to ensure that your care is person- and family-centred?
   - Probes: e.g., goal-centred, looked at the whole person, tailoring, partnering
3. Based on your experiences thus far, what kinds of changes or alterations have you needed to make to the intervention to work effectively in your community?
4. Have you experienced any overlaps with existing roles and supports available in your community? Please explain. How have you navigated these overlaps? What have you done to reduce or avoid duplication and/or promote better communication and coordination?
5. Based on your experiences implementing the CAST study to date, who are influential community members that have been important to get on board with the intervention?
6. Can you describe your working relationships with your community-based colleagues when implementing the intervention?
   - With colleagues in your organization?*(**only ask if RN-CTC is hired through an external agency/organization [i.e., via secondment])*
   - With colleagues in other organizations?
   - Can you tell me a story about a time you needed to work with others to solve a problem? Or to implement this intervention?
7. What steps have you taken to encourage other community providers to commit to engaging with you to support the intervention?
   - Which individuals have you targeted?
   - How have you approached them?
   - What information have you given them?
   - How frequently and how will you communicate with them?
8. How complicated is the CAST intervention?
   - Please consider the following aspects of the CAST intervention: duration, scope, intricacy, and number of steps involved and whether the intervention reflects a clear departure from previous practices.
9. What components of the CAST intervention have been implemented most successfully to date?

Probes:

- - care coordination, system navigation and case management (direct or indirect)
  - clinical assessments and screening
  - health education and health literacy
  - behavioural change support (e.g., behavioural activation, problem-solving therapy, supporting behaviour change)
  - clinical care (e.g., management of chronic conditions and depressive symptoms, includes medication reconciliation, review and management)
  - psychosocial and practical support
  - building trusting patient-caregiver-provider relationships
  - health promotion and disease prevention
  - self-management support
  - caregiver health and support

1. What components of the CAST intervention have been most challenging to implement?

Probes:

- - care coordination, system navigation and case management (direct or indirect)
  - clinical assessments and screening
  - health education and health literacy
  - behavioural change support (e.g., behavioural activation, problem-solving therapy, supporting behaviour change)
  - clinical care (e.g., management of chronic conditions and depressive symptoms, includes medication reconciliation, review and management)
  - psychosocial and practical support
  - building trusting patient-caregiver-provider relationships
  - health promotion and disease prevention
  - self-management support
  - caregiver health and support

1. What challenges have you faced connecting with or working with participants in implementing the intervention? How have you tried to address these?
2. Can you give an example of when the program made a difference to an older adult participant?
3. Can you give an example of when the program made a difference to a caregiver?
4. Do you have any additional comments or feedback on the implementation of the CAST study and your role to date that we have not yet discussed and you would like to add?
