# Supplementary material for: Implementation of the Community Assets Supporting Transitions (CAST) transitional care intervention for older adults with multimorbidity and depressive symptoms: A qualitative descriptive study
Source: PLoS One. 2022 Aug 5;17(8):e0271500. doi: 10.1371/journal.pone.0271500 (PMC9355229; doi:10.1371/journal.pone.0271500)
Supplement: S3 Appendix — (DOCX) [file pone.0271500.s003.docx]

**S3 Appendix. Focus Group Guides for Community Advisory Boards**

**Time Point 1**

Thank you for taking the time to participate in our focus group. I just want to start by giving you a bit of an introduction to what we will be talking about today. Today we’re going to be talking about the approaches used to plan and conduct the Community Assets Supporting Transitions (CAST) study. We are interested in how the program has been adapted to meet the needs of communities and what will need to happen to continue the program after the study is complete. We will also be talking about how patients, caregivers, and other community partners have been engaged as partners in the research team.

When we talk about engagement, we’re talking about the ways that you have participated or been involved in the work associated with the Community Assets Supporting Transitions (CAST) study or ways you could be engaged.

When we’re talking about research work, we are referring to the many different stages of CAST’s work in which you may have participated or been engaged, from the development of the partnership and research proposals, to helping us understand your community, to shaping how the study should roll out, to recruitment strategies, collecting and analyzing data, to developing key messages about the study results, and so on.

With these ideas in mind, we’d like to start with some questions about the CAST program.

1. What do you know about the CAST program and its implementation?
2. What is your understanding of why the CAST program is being implemented in your community?
3. What is the general level of receptivity in your community to implementing the CAST program?
4. Do you think the CAST program will be effective in your community?
   - Why or why not?
5. How do you feel about the CAST program being used in your community?
   - How do you feel about the plan to implement the CAST program in your community?
   - Do you have any feelings of anticipation? Stress? Enthusiasm? Why?
6. How does the CAST program compare to other similar existing programs in your community?
   - What advantages does the CAST program have compared to existing programs?
   - What disadvantages does the CAST program have compared to existing programs?
   - How will the CAST program fill current gaps?
7. Can you describe how the CAST program will be integrated into current processes?
   - How will it interact or conflict with current programs or processes?
8. What kinds of changes or alterations do you think have been made to the CAST program so it will work effectively in your community?
9. Who will decide (or what is the process for deciding) whether changes are needed to the CAST program so that it works well in your community?
   - How will you know if it is appropriate to make any changes?
10. Are there components of CAST that should not be altered?
    - Which ones should not be altered?
11. How complicated is the CAST program?
    - Please consider the following aspects of the CAST program: duration, scope, intricacy and number of steps involved, and whether the CAST program reflects a clear departure from previous practices.
12. To what extent were the needs and preferences of the individuals in your community considered when deciding to implement the CAST program?
    - Can you describe specific examples?
    - Will the CAST program be altered to meet their needs and preferences?
13. How well do you think the CAST program will meet the needs of the individuals in your community?
    - In what ways will the CAST program meet their needs? E.g. improved access to services? Reduced wait times? Help with self-management? Reduced travel time and expense?
14. How do you think the older adult study participants (e.g., patients, caregivers) in your community will respond to the CAST program?
15. What barriers will the older adult study participants in your community face to participating in the CAST program?

**Community Advisory Boards Interview – Time Point 2**

Thank you for taking the time to participate in our focus group. I just want to start by giving you a bit of an introduction to what we will be talking about today. Today we’re going to be talking about the approaches used to plan and conduct the Community Assets Supporting Transitions (CAST) study. We are interested in how the program has been adapted to meet the needs of your specific community and what will need to happen to continue the program after the study is complete. We will also be asking you about how patients, caregivers, and other community partners have been engaged as partners in the research team.

To remind you, when we talk about engagement, we’re talking about the ways that you have participated in the work associated with the Community Assets Supporting Transitions (CAST) study.

When we talk about research work, we are referring to the many different stages of the CAST study in which you may have participated or been engaged, from the development of the partnership and research proposal, to shaping how the study should roll out, to recruitment strategies, collecting and analyzing data, to developing key messages, and so on.

Next, we’re going to ask you some questions, more broadly, about the CAST program and how it has been working in your community to date.

1. What is the current stage of the research?

Probes:

- - How do you think the program is going?
  - Why do you say that?

1. Based on your understanding, has the program been implemented according to the implementation plan?

Probes:

- - [If Yes] Can you describe this?
  - [If No] Why not?

1. Do you think that this program should continue after the research project is done?
2. What, if any, aspects of the program should stay in place after the research project is done? Probes:
   - Which ones?
3. What kinds of further changes or adaptations, if any, do you think will need to be made to the program after the study ends?
4. What is the likelihood that the program will become a part of usual practice in your community? Why?

Probes:

- - How do you feel about the plan to sustain the program in your setting?
  - Do you have any feelings of anticipation? Stress? Enthusiasm? Why?

1. Since we last spoke, have you heard stories about the experiences of participants with the program?

Probes:

- - Can you describe a specific story?

1. Those are all of the questions that we have for you today. Is there anything else that you would like to share about conducting the CAST study in your community or the processes used to engage older adults and other community members as partners in the research team?
