# Supplementary material for: Implementation of the Community Assets Supporting Transitions (CAST) transitional care intervention for older adults with multimorbidity and depressive symptoms: A qualitative descriptive study
Source: PLoS One. 2022 Aug 5;17(8):e0271500. doi: 10.1371/journal.pone.0271500 (PMC9355229; doi:10.1371/journal.pone.0271500)
Supplement: S4 Appendix — (DOCX) [file pone.0271500.s004.docx]

**S4 Appendix. CAB Focus Group & Interview Participants**

| **Site** | **Time** | **Participants** |
| --- | --- | --- |
| Site A (Sudbury) | 1 | 1 Community Provider |
|  | 2 | 4 Community Providers  1 Patient and Public Research Partners |
| Site B (Hamilton) | 1 | 4 Community Providers  3 Patient and Public Research Partners |
|  | 2 | 1 Community Provider  2 Patient and Public Research Partners |
| Site C (Burlington) | 1 | 2 Community Provider  3 Patient and Public Research Partners |
|  | 2 | 2 Community Providers  2 Patient and Public Research Partners |
